# Specialization of bat-fly interactions at different elevations in a montane forest of northern Peru

**DOI:** 10.1017/S0031182025101479

**Published:** 2026-03

**Authors:** David Minaya, Juan J. Pellón, Carla Yauris, Kristhie Pillaca, Balder Choza, Jaime Pacheco, Gustavo Graciolli, José Iannacone

**Affiliations:** 1 Laboratorio de Ecología y Biodiversidad Animal, Facultad de Ciencias Naturales y Matemática, Universidad Nacional Federico Villarreal, El Agustino, Lima, Perú; 2 Laboratorio de Ecología y Conservación de Vertebrados Terrestres, Instituto de Ecología, Universidad Nacional Autónoma de México, Ciudad de México, México City, México; 3 Urb. Santo Domingo, Arequipa, Programa de Conservación de Murciélagos del Perú, Arequipa, Perú; 4 Centro de Ornitología y Biodiversidad (CORBIDI), División de Mastozoología, Surco, Lima, Perú; 5 Universidad Nacional San Luis Gonzaga, Cercado de Ica, Ica, Perú; 6 Bioacoustic Analysis Team S.A.C., Lima, Callao, Perú; 7 Setor de Zoologia, Instituto de Biociências (INBIO), Universidade Federal de Mato Grosso doSul (UFMS), Campo Grande, MS, Brazil; 8 Ciencias de la Vida, Universidad Científica del Sur, Lima, Perú

**Keywords:** altitudinal gradient, Amazonian Andes, chiroptera, ectoparasites, host-parasite interactions, network analysis, nycteribiidae, parasitism, specificity, streblidae

## Abstract

Hippoboscoidea flies exhibit highly specific ectoparasitic relationships with bats, shaped by both intrinsic factors (e.g. bat behaviour) and extrinsic factors (e.g. land use). Understanding the dynamics of these parasite–host interactions is essential for uncovering co-evolutionary patterns and informing conservation strategies. To this end, we studied bat–fly interactions across different elevations in a montane forest of Amazonas, northern Peru. The most abundant bats were *Carollia brevicauda, C. perspicillata* and *Sturnira oporaphilum*, while *Paraeuctenodes similis* and *Trichobius joblingi* were the most common flies. Most flies exhibited monoxenous host specificity. Bat–fly interaction networks revealed high modularity and specialization at both local and regional scales. Modules typically grouped bat species of the same genus or subfamily, suggesting that phylogenetic constraints and roosting behaviour may shape those interaction patterns. Nestedness within modules (compound structure) emerged in the aggregated regional network, aligning with the integrative hypothesis of specialization. Although network structures were broadly similar across sites, species turnover contributed to subtle differences in module composition and specialization. These differences were congruent with the changes in species roles of certain bats and flies. This study represents the first of its kind in Peru and addresses significant knowledge gaps in the ecology of bat–fly interactions in the Neotropics.

## Graphical abstract

**Figure:**
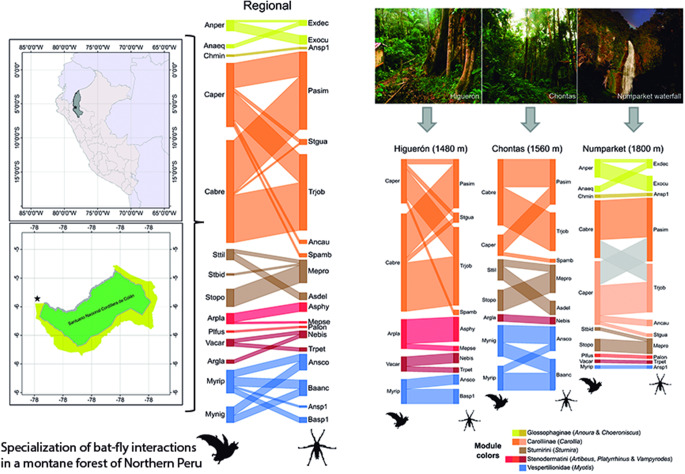

## Introduction

Bat flies (Diptera: Hippoboscoidea) are obligate, blood-feeding ectoparasites that exclusively parasitize bats and are among the most abundant and frequent hematophagous parasites in this mammalian group (Hrycyna *et al.,* 2019). These flies typically exhibit a high degree of host specificity, often associated with long-term evolutionary relationships between parasite and host lineages (Dick *et al.,* 2010). However, host specificity can also be influenced by intrinsic factors such as host behaviour, health and body size, as well as extrinsic environmental factors (Palheta *et al.,* 2020). Studying these tightly linked parasite–host interactions offers valuable insight into co-evolutionary processes and the mechanisms shaping host specialization (Dick and Patterson, 2006; Hiller *et al.,* 2021). Globally, bat flies are divided into two main families with distinct biogeographic distributions: Nycteribiidae, more diverse in the Eastern Hemisphere, and Streblidae, predominantly found in the Western Hemisphere, especially in the Neotropics (Soares *et al.,* 2013; Graciolli and Dick, 2018; Barbier *et al.,* 2019; Graciolli *et al.,* 2019). In South America, representatives of both families coexist, parasitizing a wide range of bat species (Biz *et al.,* 2023; Zapata-Mesa *et al.,* 2024). Despite their ecological relevance, detailed studies on bat–fly associations remain scarce in many parts of the Neotropics, including Peru.

Peru harbours remarkable bat diversity, with 196 species currently recorded (Pacheco *et al.,* 2021; Velazco, 2023; Diaz *et al*., 2025), of which at least 75 are known to host ectoparasites, including 66 species of bat flies (Minaya *et al.,* 2021). However, most records of bat–fly diversity in Peru are concentrated in the lowland Amazonian rainforests of Loreto and Madre de Dios (Theodor, 1967; Guerrero, 1996a; Graciolli *et al.,* 2007; Autino *et al.,* 2011; Gettinger, 2018; Gettinger *et al.,* 2020; Morales-Malacara and Guerrero, 2020). In contrast, montane forests – despite being among the most bat–diverse ecosystems in the Neotropics (Chaverri *et al.,* 2016; Bogoni *et al.,* 2021) – remain poorly studied in terms of bat ectoparasite associations. Available information from these ecosystems is sparse and typically limited to isolated records of ectoparasite presence, rather than comprehensive analyses of interactions (Biz *et al.,* 2023; Zapata-Mesa *et al.,* 2024). For example, in the montane forests of Amazonas, northern Peru, a key region within the Andean forest belt, only a single study on bat flies has been published (Ibáñez and Jara, 2008), highlighting a substantial gap in our understanding of host–parasite relationships in these high-elevation systems.

The use of ecological network analysis has become increasingly important in advancing our understanding of host–parasite systems, particularly in bat–ectoparasite relationships (Runghen *et al.,* 2021; Biz *et al.,* 2023; Zapata-Mesa *et al.,* 2024). Unlike traditional species inventories, network approaches allow researchers to explore structural properties such as modularity, nestedness and interaction specialization within ecological communities (Bezerra and Bocchiglieri, 2022). Bats and their ectoparasitic flies represent an ideal model for such studies due to their high species richness and long coevolutionary history. Network-based analyses have consistently revealed high levels of specialization and modularity in bat–fly associations, suggesting that both ecological and evolutionary factors shape these interaction patterns (Falcão *et al.,* 2022). Moreover, understanding the structure of host–parasite networks is increasingly relevant for public health, as ectoparasites may act as vectors of zoonotic pathogens, potentially facilitating transmission between wildlife and humans (Szentiványi *et al.,* 2019). Integrating network analysis into parasite–host studies therefore offers valuable insights for both ecological theory and applied conservation and health strategies.

Given this context and the need to generate local-scale data that contribute to a broader understanding of bat–ectoparasite interactions, the aim of our study was to assess the dipteran ectoparasites associated with bats in the montane forests of Amazonas, northern Peru. Specifically, we sought to examine patterns of species distribution to explore the structure of parasite–host association using interaction networks.

## Materials and methods

### Study area

The study was conducted in the hamlet of Nueva Esperanza on the Numparket Waterfall Tourist Route located in Aramango, Bagua (Amazonas, Peru). The area is characterized by low montane forest vegetation and falls within the ecoregions of Very Humid Montane Forest (Bosque Montano Muy Húmedo, BMHM) and Very Humid Premontane Forest (Bosque Premontano Muy Húmedo, BMHP) (Britto, 2017). The landscape includes both well-preserved primary forest and zones subjected to selective logging.

**Figure 1.**
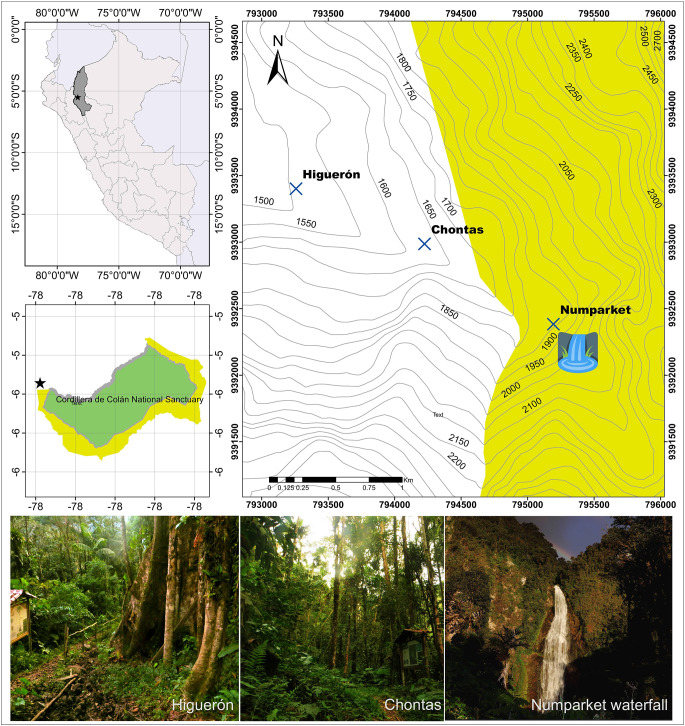
Geographic location of the sampling sites in the Amazonas region, northern Peru, where bats were captured and their ectoparasitic flies collected during the 2023–2024 field campaign. Figure 1 long description.

Fieldwork was conducted between July 2023, February 2024 and August 2024 across three sites: Numparket (1800 m a.s.l.), Chontas (1560 m a.s.l.) and Higuerón (1480 m a.s.l.). Numparket is located around the Numparket Waterfall within the conservation concession Cerro El Adobe. This area also forms part of the buffer zone of both the national sanctuary Cordillera de Colán and the communal reserve Chayu Nain (Figure 1). Due to its proximity to the waterfall and its tributary rivers, Numparket maintains high humidity throughout most of the year. The site is predominantly covered by well-preserved primary forest, with the exception of some disturbed zones near the road. In contrast, Chontas and Higuerón are located within the area of influence of Cordillera de Colán. These sites are characterized by a mosaic of preserved forest, patches of secondary growth and areas affected by selective logging. The vegetation includes species such as *Ficus paraensis, Cecropia* spp., various species of Araceae and Rubiaceae, and abundant pteridophytes (Authors’ observation).

### Bats and flies sampling

At every sampling station, 10 understory mist nets (12 × 2.5 m) spaced ∼20 m apart were used for 12 nights, distributed as three blocks of four consecutive nights (July 2023, February 2024 and August 2024) (MINAM, 2015). The nets were opened from 18:00 to 00:00 h to target species with peak foraging activity during that period (Jones *et al.,* 1996). Individuals that could not be identified in the field were collected, preserved in alcohol and deposited at the Museo Vera Alleman de la Universidad Ricardo Palma (MURP) in Lima, Peru.

All individuals were checked while alive. Bat flies were removed using entomological forceps, then fixed and preserved in polypropylene cryovials containing 70% ethanol. Specimens were cleared in 10% KOH and examined under a Nikon SMZ745 stereomicroscope for taxonomic identification using the keys of Wenzel *et al.* (1966); Wenzel (1976) and Guerrero (1993; 1994a, 1994b, 1995a, 1995b, 1996b, 1996c). Macrophotographs of external anatomy and taxonomically important structures were taken using a TOUPCAM camera mounted on a Nikon Eclipse Si microscope with a Nikon Nii LED illumination system. Image stacking was performed with ToupView software. All ectoparasite specimens were deposited in the entomological collection of the Natural History Museum, Faculty of Natural Sciences and Mathematics, Universidad Nacional Federico Villarreal (MUFV).

### Specialization of bat-fly interactions

Host specificity of bat flies was classified as follows: monoxenous (ectoparasitic flies utilizing only a single host species), oligoxenous (utilizing two or more congeneric species), pleioxenous (utilizing two or more host genera within the same family) and polyxenous (utilizing multiple hosts from different families) (Marshall, 1982; Seneviratne *et al.,* 2009). The parasite population component was analysed using standard ecological parasitological indices: prevalence (P%) and mean intensity of infection (MI), following Bush *et al.* (1997) and Bautista-Hernández *et al.* (2015).

Using the bat–fly interaction encounters, we constructed weighted bipartite networks for each of the three sites as well as an aggregated regional network. To assess the specificity of bat–fly interactions in these networks, we applied modularity and specialization metrics. We first tested for modularity (Qw) using the weighted DIRTLPAwb + algorithm (Beckett, 2016) and then evaluated low-level nestedness (within modules) using the WNODAsm metric (Pinheiro *et al.,* 2019). Following Pinheiro *et al.* (2022), we did not test for nestedness in the overall network, as all networks were significantly modular (see Results). Modularity measures the extent to which species and their interactions can be divided into subgroups (modules) that are more interconnected within themselves than with others (Newman, 2006). Nestedness reflects the pattern in which interactions of species with fewer connections (specialists) form subsets of the interactions of species with more connections (generalists) (Mariani *et al.,* 2019). Additionally, specialization was quantified using the H2’ metric (complementary specialization), which captures how selective the network is beyond what would be expected based on species relative abundances, approximated by the matrix’s marginal totals (Blüthgen *et al.,* 2006). To test the significance of these metrics, we used the equiprobable (preserving species richness and total number of interactions) and proportional (same as equiprobable but also preserving marginal sums) null models described in Pinheiro *et al.* (2022). Specifically, the restricted version of the equiprobable null model, which also maintains the modular structure during randomizations, was used to test WNODAsm, while the proportional null model was employed for *Q*_w_ and H_2_’.

Species-level metrics were used to explore variation in the specialization of bat and fly species across sites, focusing only on species present at all sites. Species-level specialization (d’) was employed to describe how selectively a bat or fly interacts with available species from the opposite group within the network, based on the frequency of their interactions (Blüthgen *et al.,* 2006). For flies, higher d′ values indicate higher host specificity. For bats, which do not choose their parasites, higher d′ values indicate that their assemblage of flies is composed of more host–specific parasites, whereas lower values indicate association with more generalist parasites. Additionally, species strength was calculated as the total sum of interaction proportions across all partners for a given species, reflecting how dependent bats or flies are on that species (Bascompte *et al.,* 2006). These species-level metrics, along with the network-level metrics mentioned above, were calculated using the package ‘bipartite’ (Dormann *et al.,* 2008) in the software R 4.4.1.

Sampling coverage of networks was also analysed following the suggestion of Chiu *et al.* (2023). This metric indicates the proportion of the total number of interaction events represented by the detected interactions. For this assessment, the ‘iNext.link’ package (Hsieh *et al.,* 2016) was used in the software R 4.4.1.

Finally, to quantify differences between interaction networks among sites, we followed the approach of Fründ (2021), which decomposes total link dissimilarity into additive components. For each pair of sites, we calculated: β_WN_, the overall dissimilarity between the two interaction networks; β_OS_, the dissimilarity attributable to changes in interactions among species shared between sites (rewiring); β_ST_, the dissimilarity attributable to species turnover, i.e. interactions that differ because one or both interacting species are present at only one site. Following Novotny (2009), β_ST_ was further partitioned into turnover caused exclusively by flies (β_ST.f_), exclusively by bats (β_ST.b_), or jointly by both (β_ST.fb_). This analysis was performed using the *betalinkr_multi* function of the package ‘bipartite’ (Dormann *et al.,* 2008) in the software R 4.4.1., specifically with the ‘commondenom’ partition method (Fründ, 2021).

## Results

### Bats and flies

A total of 160 bats were captured, including 152 individuals from the family Phyllostomidae and 8 from Vespertilionidae. The bats belonged to 23 species, of which 71 individuals from 14 species were parasitized by bat flies (Table 1). The most abundant bat species were *Carollia brevicauda* (*n* = 48), *C. perspicillata* (*n* = 36) and *Sturnira oporaphilum* (*n* = 19). The species with the highest number of parasitized individuals were *C. brevicauda* (*n* = 30) and *C. perspicillata* (*n* = 18). Among the three sampling areas, Numparket had the highest number of captured bats (*n* = 73) and parasitized individuals (*n* = 33) (Table 1). *C. brevicauda* presented the highest abundance of ectoparasites across all three sites. In Numparket and Higuerón, *C. perspicillata* ranked second in parasite abundance, while in Chontas, the second most parasitized species was *Myotis nigricans*.

**Table 1.** Bats captured along the Nueva Esperanza Trail to Numparket Falls, Amazonas, Peru, and specific characterization based on their ectoparasitic flies Table 1 long description.

	Higuerón				Chontas				Numparket			
	(1480 m)				(1560 m)				(1800 m)			
Chiroptera species	E (P)	Bf	P%	MI	E (P)	Bf	P%	MI	E (P)	Bf	P%	MI
**Pyllostomidae**												
*Anoura aequatoris*	2 (0)	0	0	–	–	–	–	–	4 (2)	2	50	1
*Anoura caudifer*	1 (0)	0	0	–	–	–	–	–	–	–	–	–
*Anoura peruana*	–	–	–	–	–	–	–	–	1 (1)	6	100	6
*Artibeus glaucus*	–	–	–	–	1 (1)	2	100	2	4 (0)	0	0	–
*Artibeus planirostris*	1 (1)	6	100	6	1 (0)	0	0	–	–	–	–	–
*Carollia brevicauda*	16 (11)	20	68.7	1.8	19 (10)	20	53	2	13 (9)	19	69.2	2.1
*Carollia perspicillata*	6 (4)	9	66.6	2.2	8 (2)	6	25	3	22 (12)	20	54.5	1.6
*Choeroniscus minor*	–	–	–	–	–	–	–	–	1 (1)	1	100	1
*Desmodus rotundus*	3 (0)	0	0	–	3 (0)	0	0	–	2 (0)	0	0	–
*Platyrrhinus fusciventris*	–	–	–	–	–	–	–	–	1 (1)	1	100	1
*Platyrrhinus umbratus*	–	–	–	–	1 (0)	0	0	–	1 (0)	0	0	–
*Sturnira bidens*	1 (0)	0	0	–	2 (0)	0	0	–	3 (1)	1	33.3	1
*Sturnira erythromos*	1 (0)	0	0	–	2 (0)	0	0	–	3 (0)	0	0	–
*Sturnira magna*	–	–	–	–	–	–	–	–	2 (0)	0	0	–
*Sturnira oporaphilum*	2 (0)	0	0	–	5 (2)	6	40	3	12 (4)	4	33.3	1
*Sturnira tildae*	–	–	–	–	4 (2)	6	50	3	–	–	–	–
*Vampyressa thyone*	1 (0)	0	0	–	–	–	–	–	–	–	–	–
*Vampyrodes caraccioli*	1 (1)	3	100	3	–	–	–	–	2 (1)	1	50	1
**Vespertilionidae**												
*Eptesicus chiriquinus*	1 (0)	0	0	–	–	–	–	–	–	–	–	–
*Myotis caucensis*	1 (0)	0	0	–	–	–	–	–	–	–	–	–
*Myotis nigricans*	–	–	–	–	2 (2)	9	100	4.5	1 (0)	0	0	–
*Myotis riparius*	1 (1)	5	100	5	1 (1)	7	100	7	1 (1)	1	100	1
Total	38 (18)	43	46.1	2.3	49 (20)	56	40.8	2.8	73 (33)	56	45.2	1.7

Bf, bat fly abundance; E(P), no. of examined bats (parasitized); MI, mean intensity; P%, prevalence; S, bat fly species richness.

**Figure 2.**
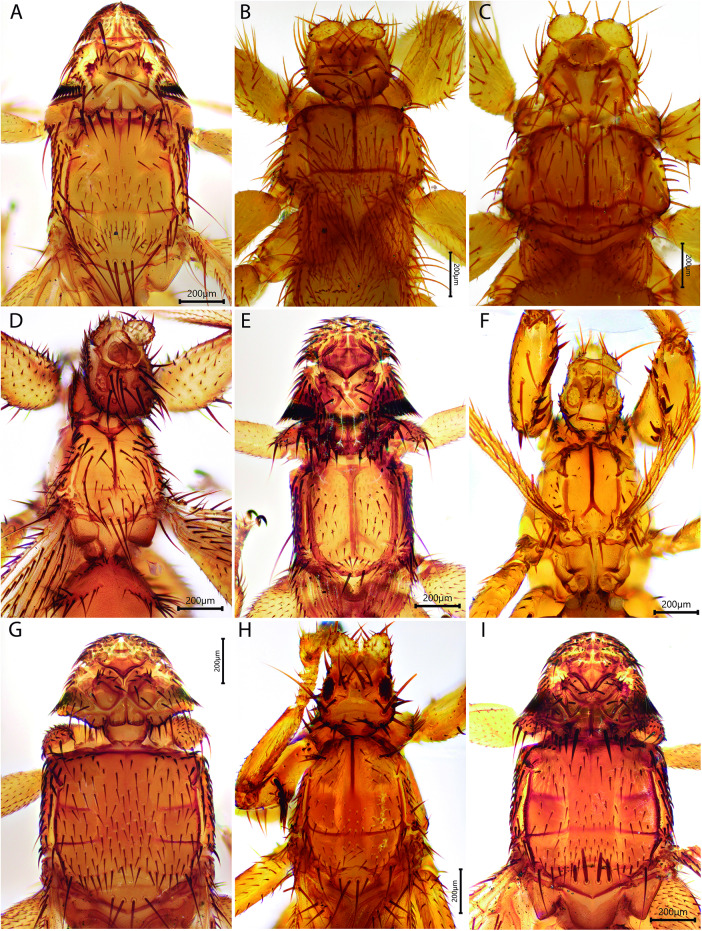
Species of ectoparasitic diptera from bats captured along the Nueva Esperanza Trail to Numparket Falls, Amazonas, Peru (first part). (A) *Anastrebla caudiferae*, (B) *Aspidoptera falcata*, (C) *Exastinion oculatum*, (D) *Megistopoda proxima*, (E) *Metelasmus pseudopterus*, (F) *Neotrichobius bisetosus*, (G) *Paraeuctenodes similis*, (H) *Paratrichobius longicrus complex*, (I) *Strebla guajiro*. Figure 2 long description.

**Figure 3.**
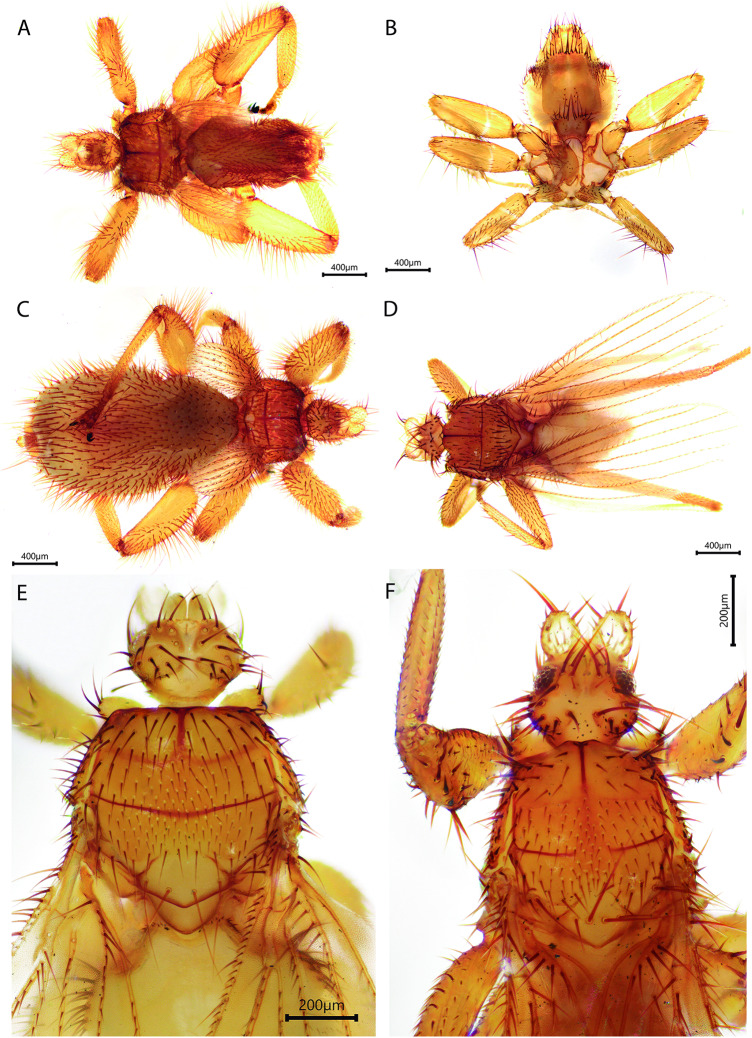
Species of ectoparasitic diptera from bats captured along the Nueva Esperanza Trail to Numparket Falls, Amazonas, Peru (second part). (A) *Anatrichobius scorzai*, (B) *Basilia anceps*, (C) *Anatrichobius* sp., (D) *Speiseria ambigua*, (E) *Trichobius joblingi*, (F) *Paratrichobius salvini complex*. Figure 3 long description.

A total of 155 ectoparasitic flies were collected, representing 19 species from the families Streblidae (17 species) and Nycteribiidae (2 species) (Figures 2 –3). The most abundant fly species were *Paraeuctenodes similis* Wenzel, 1976 (*n* = 44), *Trichobius joblingi* Wenzel, 1966 (*n* = 43) and *Megistopoda proxima* (Séguy, 1926) (*n* = 13). Numparket and Higuerón exhibited the highest bat fly species richness (*s* = 11) and abundance (*n* = 56), followed by Chontas (*s* = 8, *n* = 43). Only *P. similis* and *T. joblingi* were recorded in all three areas. In terms of host specificity, most bat fly species were classified as monoxenous (*n* = 10), followed by oligoxenous (*n* = 8), and pleioxenous (*n* = 1) (Table 2).

**Table 2.** Associations and characterization of ectoparasitic flies from bat captured along the Nueva Esperanza Trail to Numparket Falls, Amazonas, Peru Table 2 long description.

Diptera species	Chiroptera species	H	C	N	Spe
**Nycterebiidae**					
Basilia anceps*					Oli
	*Myotis nigricans*	–	4	–	
	*Myotis riparius*	–	5	–	
*Basilia* sp. (*group juquiensis*)					Mon
	*Myotis riparius*	3	–	–	
**Streblidae**					
Anastrebla caudiferae**					Oli
	*Carollia perspicillata*	–	–	1	
	*Carollia brevicauda*	1	–	–	
*Anastrebla* sp.					Mon
	*Choeroniscus minor*	–	–	1	
Anatrichobius scorzai*					Oli
	*Myotis nigricans*	–	5	–	
	*Myotis riparius*	2	2	–	
*Anatrichobius* sp.					Mon
	*Myotis riparius*	–	–	1	
Aspidoptera falcata*					Mon
	*Sturnira tildae*	–	4	–	
Aspidoptera phyllostomatis*					Mon
	*Artibeus planirostris*	–	–	5	
Exastinion deceptivum**					Oli
	*Anoura aequatoris*	–	–	2	
	*Anoura peruana*	–	–	1	
Exastinion oculatum**					Mon
	*Anoura peruana*	–	–	5	
Megistopoda proxima*					Oli
	*Sturnira bidens*	–	–	1	
	*Sturnira oporaphilum*	–	6	4	
	*Sturnira tildae*	–	2	–	
Metelasmus pseudopterus*					Mon
	*Artibeus planirostris*	1	–	–	
Neotrichobius bisetosus*					Ple
	*Artibeus glaucus*	–	2	–	
	*Vampyrodes caraccioli*	2	–	–	
Paraeuctenodes similis**					Oli
	*Carollia brevicauda*	9	8	13	
	*Carollia perspicillata*	1	5	8	
Paratrichobius longicrus complex*					Mon
	*Platyrrhinus fusciventris*	–	–	1	
Speiseria ambigua*					Mon
	*Carollia perspicillata*	1	1	–	
Strebla guajiro*					Oli
	*Carollia brevicauda*	1	–	–	
	*Carollia perspicillata*	1	–	1	
Trichobius joblingi*					Oli
	*Carollia brevicauda*	9	12	6	
	*Carollia perspicillata*	6	–	10	
Paratrichobius salvini complex**					Mon
	*Vampyrodes caraccioli*	1	–	1	
**Abundance**		**43**	**56**	**56**	
**Richness**		**11**	**8**	**11**	

C, Chontas (1560 m); H, Higuerón (1480 m); N, Numparket (1800 m); P%, Prevalence; Spe, Specificity (Mon, Monoxenous; Oli, Oligoxenous; Ple, Pleioxenous).

** New species record for Peru; *New record only for Amazonas.

### Specialization of bat–fly interactions

**Figure 4.**
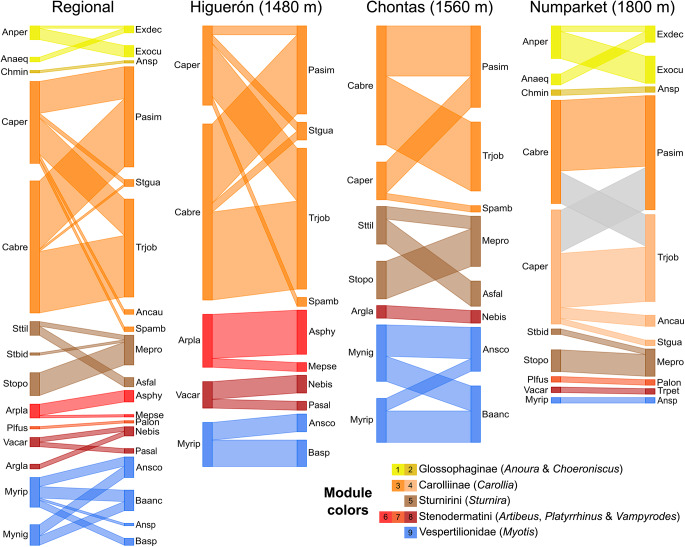
Modular structure of bat–fly interaction networks along the Nueva Esperanza Trail to Numparket Falls, Amazonas, Peru. The regional network is the result of the aggregation of interactions of the three other local networks. Interactions and species of the same module share specific colours and interactions between species of different modules are in grey. Figure 4 long description.

Abbreviations: *Anoura aequatoris* = Anaeq, *Anoura peruana* = Anper, *Artibeus glaucus* = Argla, *Artibeus planirostris* = Arpla, *Carollia brevicauda* = Cabre, *Carollia perspicillata* = Caper, *Choeroniscus minor* = Chmin, *Platyrrhinus fusciventris* = Plfus, *Sturnira bidens* = Stbid, *Sturnira oporaphilum* = Stopo, *Sturnira tildae* = Sttil, *Vampyrodes caraccioli* = Vacar, *Myotis nigricans* = Mynig, *Myotis riparius* = Myrip. *Basilia anceps* = Baanc, *Basilia* sp = Basp, *Anastrebla caudiferae* = Ancau, *Anastrebla* sp = Ansp, *Anatrichobius scorzai* = Ansco, *Anatrichobius* sp = Ansp, *Aspidoptera falcata* = Asfal, *Aspidoptera phyllostomatis* = Asphy, *Exastinion deceptivum* = Exdec, *Exastinion oculatum* = Exocu, *Megistopoda proxima* = Mepro, *Metelasmus pseudopterus* = Mepse, *Neotrichobius bisetosus* = Nebis, *Paraeuctenodes similis* = Pasim, *Paratrichobius longicrus* = Palon, *Paratrichobius salvini* = Pasal, *Speiseria ambigua* = Spamb, *Strebla guajiro* = Stgua, *Trichobius joblingi* = Trjob.

Sampling coverage of bat–fly networks was always above 0.85 (Table 3), indicating that they are a good representation of bat–fly interactions at each site as well as at the regional scale. All networks exhibited a modular topology, but nestedness within modules was observed only in the regional network (Table 3). Specificity of interactions was intermediate to high across all networks, as indicated by Qw (≥0.48) and H2’ (≥0.75). The highest values for these metrics were observed in Chontas (middle elevation), while Higuerón and Numparket showed similar values, still reflecting high specificity. The modular structure showed a clear partition based on the phylogenetic relationships of bats, with modules never including unrelated taxa of bats (Figure 4). *Anoura, Carollia, Myotis* and *Sturnira* species were always grouped within the same module, except for *Carollia* at higher altitudes (Numparket). In this latter case, *C. brevicauda* and *C. perspicillata* formed their own module, although they still shared the same flies.

**Table 3.** Sampling coverage and structural properties of bat–fly interaction networks along the Nueva Esperanza Trail to Numparket Falls, Amazonas, Peru. The regional network is the result of the aggregation of interactions of the three local networks Table 3 long description.

Network	SC	Q_w_	WNODA_sm_	H_2_’
Regional	0.96	**0.59**	**60.74**	**0.75**
Higuerón (1480 m)	0.86	**0.51**	70	**0.75**
Chontas (1560 m)	0.98	**0.66**	31.25	**0.92**
Numparket (1800 m)	0.88	**0.48**	75	**0.65**

SC, sampling coverage; Qw, weighted modularity; WNODA_sm_, within-module nestedness, H2’: complementary specialization. Statistically significant values (*P* < 0.05) are in bold.

**Figure 5.**
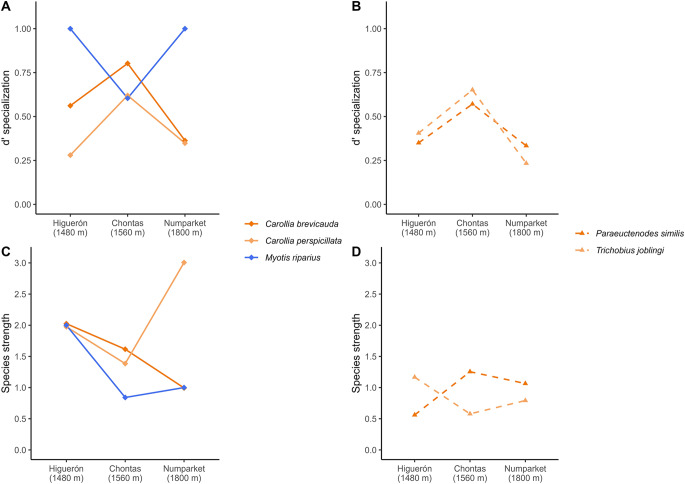
Species-level metrics of bat–fly interaction networks along the Nueva Esperanza Trail to Numparket Falls, Amazonas, Peru. Species-level specialization d’ (A and B) and species strength (C and D). Only species present in the three evaluated sites are assessed. Figure 5 long description.

Species showed different patterns across sites in terms of species-level specialization and species strength (Figure 5, Supplementary table S1-S2). *Carollia* species exhibited higher specialization at middle elevations (Chontas), with *C. brevicauda* consistently showing higher values than *C. perspicillata. Myotis riparius* displayed the highest specialization values at lower (Higuerón) and higher (Numparket) elevations. However, species strength for *C. brevicauda* decreased continuously from lower to higher elevations, while values for *C. perspicillata* increased at higher elevations, eventually surpassing *C. brevicauda. Myotis riparius* showed the same pattern observed in specialization, with lower species strength values at middle elevations. Among flies, *T. joblingi* was more specialized than *P. similis* at lower and middle elevations, but roles reversed at higher elevations. However, *T. joblingi* had higher species strength values than *P. similis* at the lower-elevation site, while the opposite occurred at middle- and higher-elevation sites.

Network dissimilarity between elevations was moderate to high, with β_WN_ values ranging from 0.60 (Higuerón–Chontas) to 0.70 (Chontas–Numparket) (Table 4). Across all pairwise comparisons, the contribution of rewiring among shared species (β_OS_) was consistently low (0.03–0.04), representing only 5–7% of total link dissimilarity. In contrast, species turnover (β_ST_) accounted for the vast majority of network differences (93–95%). Within the turnover component, co-turnover of both bats and flies (β_ST.fb_) was consistently the dominant factor, contributing approximately 50–60% of β_ST_ in all comparisons. Turnover restricted to flies (β_ST.f_) also made a substantial contribution (33–40%), whereas turnover restricted to bats (β_ST.b_) was smaller (0–21%) and, in one comparison, absent.

**Table 4.** Pairwise network dissimilarity between sites. Β_OS_ represents the dissimilarity attributable to rewiring among species shared between sites. Β_WN_ is the overall dissimilarity between interaction networks. Β_ST_ corresponds to the component of dissimilarity explained by species turnover. Β_ST.F_, β_ST.B_ and β_ST.Fb_ indicate the portions of β_ST_ attributable to turnover restricted to fly species, restricted to bat species, and jointly to both trophic levels, respectively Table 4 long description.

Networks	β_OS_	β_WN_	β_ST_	β_ST.f_	β_ST.b_	β_ST.fb_
Higueron – Chontas	0.04	0.6	0.56	0.16	0.12	0.28
Higueron – Numparket	0.04	0.57	0.54	0.21	0	0.32
Chontas – Numparket	0.04	0.7	0.67	0.22	0.07	0.37

## Discussion

### Bats and flies

This study expands the current knowledge of bat–ectoparasite interactions in the montane forests of northern Peru by documenting 19 species of parasitic flies associated with 14 bat species. All bat fly species reported represent new records for Amazonas (Minaya *et al.,* 2021), and *Anastrebla caudiferae, Exastinion deceptivum, Exastinion oculatum, P. similis* and the *Paratrichobius salvini* complex are documented for the first time in Peru. An important observation concerns the record of *T. joblingi*. Although this species was previously reported from Condorcanqui, Amazonas, by Ibáñez and Jara (2008), we noted inconsistencies between their figure and the diagnostic characters of *T. joblingi*. Based on our specimens, we provide the first confirmed record of *T. joblingi* for the department of Amazonas.

Two bat fly species, *P. similis* and *T. joblingi*, represented the dominant and most abundant core ectoparasites in the bats sampled in Amazonas. Both species parasitized more than 50% of the *C. brevicauda* and *C. perspicillata* individuals examined. A similar pattern was observed in the Magdalena River basin (López-Rivera *et al.,* 2024) and in Caldas (Raigosa *et al.,* 2020), both in Colombia, where approximately 50% of individuals were primarily parasitized by *T. joblingi* and *P. similis*, consistent with our observations in Amazonas. These findings suggest that both dipteran species exhibit strong host specificity toward *Carollia* bats, maintaining a stable host–parasite association across distinct Neotropical ecosystems. This stability may be further reinforced by the high sociality and frequent sharing of roosts and foraging resources among *Carollia* bats, which facilitate parasite transmission (Altizer *et al.,* 2003; McLellan and Koopman, 2008; Rifkin *et al.,* 2012; Webber *et al.,* 2015; Medina and Torres, 2018).

Among other streblid flies recorded, *M. proxima* and *N. bisetosus* stand out for exhibiting the broadest host associations, though for distinct biological reasons. *M. proxima* was the only species parasitizing more than one congeneric host within *Sturnira*, whereas *N. bisetosus* exploited two hosts from different genera – *A. glaucus* and *V. caraccioli*. Under classical host-specificity categories (Seneviratne *et al.,* 2009), *M. proxima* qualifies as oligoxenous and *N. bisetosus* as pleioxenous, the latter being an uncommon pattern in Streblidae, a group known for strong phylogenetic fidelity (Dick and Patterson, 2006; Autino *et al.,* 2011).

These broader host associations likely reflect ecological opportunities for cross–host transmission. Although direct evidence for multispecies roost sharing among *Sturnira* in Andean forests is limited, phyllostomid bats commonly use diverse natural shelters, where mixed-species roosts can occur (Kunz and Lumsden, 2003; Patterson *et al.,* 2007). Such conditions plausibly increase contact opportunities among sympatric hosts and may facilitate the movement of *M. proxima* among closely related *Sturnira* species. Similarly, rare pleioxenous patterns like that of *N. bisetosus* have been reported in other Neotropical systems (e.g. *Neotrichobius delicatus* in Loreto, Peru; Autino *et al.,* 2011), typically involving flies associated with ecologically overlapping phyllostomid bats (Fagundes *et al.,* 2017).

Host–parasite associations between bat flies and their chiropteran hosts are generally characterized by strong specificity, as seen in *Basilia* and *Anatrichobius*, which primarily parasitize *Myotis* species (Guerrero, 1995b; Ospina-Pérez *et al.,* 2023), or *Exastinion*, apparently restricted to *Anoura* (Guerrero, 1995b). Similar patterns have been documented in Peru (Minaya *et al*., 2021), and our findings corroborate these associations while extending their known geographic distributions into the montane forests of northern Peru.

### Specialization of bat–fly interactions

This study indicates a great specialization at the community level among parasitic bat–flies and their hosts in the montane forests near Nueva Esperanza, Amazonas. These specific associations are well documented in different parts of the world (Lim *et al.,* 2020; Poon *et al.,* 2023); the Neotropical region (Guerrero, 2019; Ospina-Pérez *et al.,* 2023; Ramírez-Martínez and Tlapaya-Romero, 2023; França *et al.,* 2024) and specifically Peru are no exception (Autino *et al.,* 2011).

Bat–fly interaction networks showed high specificity (high Qw and H2’), a pattern frequently observed in bat–fly interactions at other locations (Fagundes *et al.,* 2017; Urbieta *et al*., 2022; Hiller *et al.,* 2021; Ramalho *et al.,* 2021; Ospina-Pérez *et al.,* 2023; Ramírez-Martínez and Tlapaya-Romero, 2023). This high specificity is mainly driven by the parasitic nature of these interactions, where parasites typically depend strongly on specific hosts to maximize their fitness (Runghen *et al.,* 2021). Such a high degree of dependency often results in parasite–host networks forming modules composed of phylogenetically related species (Felix *et al.,* 2022), as was also observed in all our bat–fly networks. The regional network showed internally nested modules due to the aggregation of interactions that were uniquely observed at specific sites. For example, this pattern is evident in the module of *Myotis* species: at Higuerón, only *M. riparius* is present, interacting with *A. scorzai* and *Basilia* sp.; at Chontas, *M. nigricans* appears along with *B. anceps*; and at Numparket, *Anatrichobius* sp. is present. When aggregating all these interactions, nestedness increases within the *Myotis* module, and a similar pattern occurs in other modules, resulting in a compound structure (internally nested modules). This is consistent with the integrative hypothesis of specialization proposed for parasitic networks, which suggests that at larger scales, internally nested modules are more likely to emerge due to the aggregation of different allopatric species and interactions (Felix *et al.,* 2022).

In addition to the evolutionary component behind interactions between bats and flies, these associations have also been particularly discussed in the context of roost-sharing among bat species (Reckardt and Kerth, 2006; Patterson *et al.,* 2007; Fagundes *et al.,* 2017; Urbieta *et al.,* 2022). Logically, species that share roosts are more susceptible to sharing flies, as mentioned in the previous section. This could also be a factor driving the interactions observed in this study, although specific information on roost-sharing is not available for most species. Various bat species share roost with congeneric species, especially in caves (Tanalgo *et al.,* 2022), which could have contributed to the independent module aggregation observed in our study for *Anoura, Carollia, Myotis* and *Sturnira*, which have been reported to roost in caves frequently (Tanalgo *et al.,* 2022). The Stenodermatini species recorded in our study apparently prefer different kinds of roosts (Garbino and Tavares, 2018), and as far as we know, there are no records of them roosting together. However, *A. glaucus* and *V. caraccioli* were both hosts of *P. salvini complex. Artibeus glaucus* is a strictly tent-making bat (Ortega *et al.,* 2015), and *V. caraccioli* is also suggested to be a tent-making bat (Page and Dechmann, 2022). This may suggest they could potentially share roosts; however, tents are usually inhabited by only one species (Rodríguez-Herrera *et al.,* 2007). Nevertheless, considering that bats can colonize a tent previously used (Rodríguez-Herrera *et al.,* 2007) or may possibly try to exclude bats from an existent tent (Kunz and McCracken, 1996), there is a possibility that flies can be transmitted through tents. This could represent a strategy by flies to spread among populations and species, taking advantage of the complex roosting dynamics of tent-making bats (Chaverri and Kunz, 2006, 2010; Fernandez *et al.,* 2021). In summary, roosting behaviour of bats may be closely related to their interactions with flies and should be explored in detail to better understand these relationships.

Specialization and species strength of species varied across sites but followed different patterns, which may indicate that bat–fly relationships change according to specific properties of each site, even though the overall network structure can remain similar (Nielsen and Totland, 2014). The observed changes in specialization and species strength for both *Carollia* species, *T. joblingi* and *P. similis*, are consistent with the module separation observed for *Carollia* at Numparket. At this site, fly species associated with the genus *Carollia* were much more dependent on *C. perspicillata* (as indicated by a disproportionately high species strength), while the specialization of *P. similis* at this site surpassed that of *T. joblingi*. Although these metrics do not causally drive modularity, they highlight complementary information about how these species structure the network. This suggests that not only species turnover or richness differences can modify networks, but also that changes in how bat and fly species interact can be a driver of subtle network structural variations (Jordán *et al.,* 2008; Fründ, 2021).

Despite of the evident species-level variations, rewiring contributed only a small fraction of total dissimilarity in all pairwise comparisons, indicating that species occurring at multiple sites tended to retain similar partners. In contrast, species turnover accounted for more than 90% of link dissimilarity, with the largest contribution coming from the joint turnover of bats and flies, followed by turnover restricted to flies. This pattern reflects both the inherent specificity of flies (Runghen *et al.,* 2021) and the considerable variation in bat and fly assemblages across elevations. These results show that differences in community composition were the main driver of the to the observed variation in interactions, although network structure remained broadly similar among sites as has been observed in other studies (e.g. Kemp *et al.,* 2017; White *et al.,* 2022).

In conclusion, we provide novel insights into the diversity and structure of bat–fly interactions in the montane forests of northern Peru and represents the first in the country to apply a network-based approach to these associations. Our records reveal new distributional records at both local and national levels. Bat–fly relationships were highly specialized at both local and regional scales, with slight structural variation across sites. Network structure appears to be shaped by phylogenetic constraints and the roosting behaviour of bat hosts. Species turnover was the major factor behind interaction differences along the elevational gradient. However, species-level roles of bats and flies varied across sites, suggesting that specific interaction dynamics, rather than species turnover alone, contributed to the observed differences in interactions. This also points to a possible interplay between environmental factors and bat–fly relationships. Overall, our findings in this important but previously unexplored region of the Peruvian Andes contribute substantially to the broader ecological understanding of bat–fly interactions in Neotropical ecosystems.

## Supporting information

10.1017/S0031182025101479.sm001 Minaya et al. supplementary material Minaya et al. supplementary material

## Supplementary material

The supplementary material for this paper can be found at https://doi.org/10.1017/S0031182025101479.

## Figure 1 Long description

The composite image includes several elements related to sampling sites in northern Peru. The top left map shows the location of the sites within Peru, highlighting the Cordillera de Colán National Sanctuary. The middle map details the topography around Higuerón, Chontas and Numparket, with contour lines indicating elevation levels from 1480 m to 2150 m. Numparket is marked near a waterfall symbol. The bottom row contains photographs of the three sites: Higuerón, Chontas and the Numparket waterfall, each showing dense forest vegetation. The maps and photos illustrate the geographic and environmental context of the sampling sites, emphasizing their location within a preserved forest area and proximity to the Numparket waterfall.

Navigate back to Figure 1.

## Figure 2 Long description

The image A showing an amber-brown insect body in dorsal view with dense dark bristles across the head and thorax. Two lateral appendages extend outward from the mid-body. A vertical scale bar at the lower right reads “200 µm”. The image B showing an amber-brown insect body in dorsal view with a rounded head region and many bristles. The thorax is broad with darker internal lines. A vertical scale bar at the lower right reads “200 µm”. The image C showing an amber-brown insect body in dorsal view with a rounded head and prominent bristles along the margins. The thorax has darker linear markings. A vertical scale bar at the lower right reads “200 µm”. The image D showing an amber-brown insect body with the head near the top and multiple appendages extending laterally. Bristles are distributed across the body and along the appendages. A vertical scale bar at the lower right reads “200 µm”. The image E showing an amber-brown insect body in dorsal view with a darker, banded area across the upper thorax and dense bristles around the head. Two lateral appendages extend outward. A vertical scale bar at the lower right reads “200 µm”. The image F showing an amber-brown insect body with multiple appendages raised upward and outward, including two large appendages near the top corners. The body midline is marked by darker internal lines and bristles are present across the body. A vertical scale bar at the lower right reads “200 µm”. The image G showing an amber-brown insect body in dorsal view with a broad thorax and dense bristles, especially along the upper margin. The head region is rounded with darker markings. A vertical scale bar at the lower right reads “200 µm”. The image H showing an amber-brown insect body with appendages extending upward and outward from both sides. The thorax is elongated with darker internal lines and scattered bristles. A vertical scale bar at the lower right reads “200 µm”. The image I showing an amber-brown insect body in dorsal view with a rounded head region and dense bristles along the upper margin. The thorax is broad with darker internal lines. A vertical scale bar at the lower right reads “200 µm”.

Navigate back to Figure 2.

## Figure 3 Long description

The image A showing an orange-brown insect specimen on a white background, oriented diagonally with the head toward the left. Multiple legs extend outward and fine hair-like structures cover the body and legs. A black scale bar with the text “400 µm” is placed near the lower right. The image B showing an orange-brown insect specimen on a white background, oriented with the head toward the top. Legs extend symmetrically to the left and right. Fine hair-like structures are present along the body margins and legs. A black scale bar with the text “400 µm” is placed near the lower left. The image C showing an orange-brown insect specimen on a white background, oriented with the head toward the right. The body is broader and densely covered with hair-like structures, with legs extending from the sides. A black scale bar with the text “400 µm” is placed near the lower left. The image D showing an orange-brown insect specimen on a white background, oriented with the head toward the left. Two long, narrow, translucent wings extend to the right, with multiple visible wing veins. Legs extend beneath the body. A black scale bar with the text “400 µm” is placed near the lower right. The image E showing a closer view of an orange-brown insect body region on a white background. The central body area has many hair-like structures and several longer bristles along the sides. A black scale bar with the text “200 µm” is placed near the lower right. The image F showing a closer view of an orange-brown insect body region on a white background, including the head and upper body with prominent bristles and hair-like structures. A leg segment is visible along the left side. A vertical black scale bar with the text “200 µm” is placed near the upper right.

Navigate back to Figure 3.

## Figure 4 Long description

The diagram presents modular structures of bat-fly interaction networks across four locations: Regional, Higuerón (1480 m), Chontas (1560 m) and Numparket (1800 m). Each location is represented by a column with connections between species. The modules are color-coded: yellow for Glossophaginae (Anoura and Choeroniscus), orange for Carolliinae (Carollia), brown for Sturnirini (Sturnira), red for Stenodermatini (Artibeus, Platyrrhinus and Vampyrodes) and blue for Vespertilionidae (Myotis). The connections illustrate interactions between species within and across these modules.

Navigate back to Figure 4.

## Figure 5 Long description

The image A showing a multi-line graph titled with the panel label “A”. Vertical axis label: “d specialization”. Horizontal axis categories: “Higuerón (1400 m)”, “Chontas (1560 m)”, “Numparket (1800 m)”. Vertical axis range: 0.00 to 1.00. Legend labels: “Carollia brevicauda”, “Carollia perspicillata”, “Myotis riparius”. Line styling: three solid lines with point markers. Data points as coordinate pairs: Carollia brevicauda: (Chontas (1560 m), 0.80), (Numparket (1800 m), 0.35). Carollia perspicillata: (Higuerón (1400 m), 0.25), (Chontas (1560 m), 0.60), (Numparket (1800 m), 0.35). Myotis riparius: (Higuerón (1400 m), 1.00), (Chontas (1560 m), 0.60), (Numparket (1800 m), 1.00). Notable highs and lows from the plotted values: Myotis riparius reaches 1.00 at Higuerón (1400 m) and 1.00 at Numparket (1800 m) and 0.60 at Chontas (1560 m). Carollia perspicillata reaches 0.60 at Chontas (1560 m) and 0.25 at Higuerón (1400 m). Carollia brevicauda includes 0.80 at Chontas (1560 m) and 0.35 at Numparket (1800 m). The image B showing a multi-line graph titled with the panel label “B”. Vertical axis label: “d specialization”. Horizontal axis categories: “Higuerón (1400 m)”, “Chontas (1560 m)”, “Numparket (1800 m)”. Vertical axis range: 0.00 to 1.00. Legend labels: “Paratrichobius similis” and “Trichobius joblingi”. Line styling: two dashed lines with point markers. Data points as coordinate pairs: Paratrichobius similis: (Higuerón (1400 m), 0.40), (Chontas (1560 m), 0.60), (Numparket (1800 m), 0.30). Trichobius joblingi: (Higuerón (1400 m), 0.35), (Chontas (1560 m), 0.65), (Numparket (1800 m), 0.25). Notable highs and lows from the plotted values: Trichobius joblingi reaches 0.65 at Chontas (1560 m) and 0.25 at Numparket (1800 m). Paratrichobius similis reaches 0.60 at Chontas (1560 m) and 0.30 at Numparket (1800 m). The image C showing a multi-line graph titled with the panel label “C”. Vertical axis label: “Species strength”. Horizontal axis categories: “Higuerón (1400 m)”, “Chontas (1560 m)”, “Numparket (1800 m)”. Vertical axis range: 0.0 to 3.0. Legend labels: “Carollia brevicauda”, “Carollia perspicillata” and “Myotis riparius”. Line styling: three solid lines with point markers. Data points as coordinate pairs: Carollia brevicauda: (Higuerón (1400 m), 2.00), (Chontas (1560 m), 1.50), (Numparket (1800 m), 1.00). Carollia perspicillata: (Higuerón (1400 m), 2.00), (Chontas (1560 m), 1.30), (Numparket (1800 m), 3.00). Myotis riparius: (Higuerón (1400 m), 2.00), (Chontas (1560 m), 0.80), (Numparket (1800 m), 1.00). Notable highs and lows from the plotted values: Carollia perspicillata reaches 3.00 at Numparket (1800 m) and 1.30 at Chontas (1560 m). Myotis riparius reaches 0.80 at Chontas (1560 m) and 2.00 at Higuerón (1400 m). Carollia brevicauda includes 2.00 at Higuerón (1400 m) and 1.00 at Numparket (1800 m). The image D showing a multi-line graph titled with the panel label “D”. Vertical axis label: “Species strength”. Horizontal axis categories: “Higuerón (1400 m)”, “Chontas (1560 m)”, “Numparket (1800 m)”. Vertical axis range: 0.0 to 3.0. Legend labels: “Paratrichobius similis” and “Trichobius joblingi”. Line styling: two dashed lines with point markers. Data points as coordinate pairs: Paratrichobius similis: (Higuerón (1400 m), 1.00), (Chontas (1560 m), 0.50), (Numparket (1800 m), 0.70). Trichobius joblingi: (Higuerón (1400 m), 0.50), (Chontas (1560 m), 1.00), (Numparket (1800 m), 0.90). Notable highs and lows from the plotted values: Paratrichobius similis reaches 1.00 at Higuerón (1400 m) and 0.50 at Chontas (1560 m). Trichobius joblingi reaches 1.00 at Chontas (1560 m) and 0.50 at Higuerón (1400 m). Across A to D, the plotted values show changes across the three horizontal categories for each legend label, with several series peaking at Chontas (1560 m) in image B and peaking at Numparket (1800 m) for Carollia perspicillata in image C and Myotis riparius reaching 1.00 at both Higuerón (1400 m) and Numparket (1800 m) in image A.

Navigate back to Figure 5.

## Table 1 Long description

The table reports, for each bat species at three sites (Higuerón 1480 m, Chontas 1560 m, Numparket 1800 m), the number examined and parasitized, total bat flies counted, prevalence (percent parasitized), and mean intensity (flies per parasitized bat). Overall totals show 38 bats examined at Higuerón (18 parasitized; 43 flies; 46.1% prevalence; mean intensity 2.3), 49 at Chontas (20 parasitized; 56 flies; 40.8%; intensity 2.8), and 73 at Numparket (33 parasitized; 56 flies; 45.2%; intensity 1.7). Carollia brevicauda and Carollia perspicillata contribute the largest sample sizes and fly counts at all sites, with prevalence generally around half to two-thirds (e.g., C. brevicauda 53–69.2% and C. perspicillata 25–66.6%) and mean intensities near 1.6–3.0. Several species show 100% prevalence but are based on single or very small samples, such as Anoura peruana at Numparket (1 examined, 6 flies, intensity 6) and Myotis riparius at all sites (each 1 examined and parasitized; intensities 5, 7, and 1). Some species had no flies detected where sampled, including Desmodus rotundus at all sites (0% prevalence) and multiple Sturnira species at Higuerón and Chontas. Comparatively, Numparket has the highest number of bats examined but a lower average fly load per parasitized bat than the other sites, suggesting more widespread but lighter infestations there. Dashes indicate species not captured at a site, and results for rare species should be interpreted cautiously due to very small sample sizes.

Navigate back to Table 1.

## Table 2 Long description

Counts of ectoparasitic bat flies are listed by fly species and their bat host species, with separate totals for three sampling sites (H, C, N) and a host-specificity category (monoxenous, oligoxenous, or pleioxenous). Overall abundance was similar at C and N (56 each) and lower at H (43). Taxon richness was highest at H and N (11 taxa each) and lowest at C (8). The largest infestations involved Carollia bats: Paraeuctenodes similis reached 9/8/13 on Carollia brevicauda and 1/5/8 on Carollia perspicillata (H/C/N), and Trichobius joblingi reached 9/12/6 on C. brevicauda and 6/0/10 on C. perspicillata. Several associations were site-restricted, such as Aspidoptera phyllostomatis on Artibeus planirostris only at N (5) and Exastinion oculatum on Anoura peruana only at N (5). Myotis hosts carried multiple fly taxa, including Basilia anceps at C (4–5) and Anatrichobius scorzai at C and H (up to 5 at C). Specificity varies across taxa, with many monoxenous species but several oligoxenous species concentrated on Carollia and Sturnira hosts. Dashes indicate no individuals recorded for that host-site combination, so absence may reflect sampling limits rather than true absence.

Navigate back to Table 2.

## Table 3 Long description

The table reports four network metrics for a regional bat–fly interaction network and three local networks at different sites: sampling coverage (SC), weighted modularity (Qw), within-module nestedness (WNODAsm), and complementary specialization (H2’). Sampling coverage is high overall, ranging from 0.86 at Higuerón to 0.98 at Chontas; the regional network is 0.96. Modularity varies from 0.48 at Numparket to 0.66 at Chontas, with the regional value at 0.59. Within-module nestedness is lowest at Chontas (31.25) and highest at Numparket (75), with Higuerón at 70 and the regional network at 60.74. Specialization is highest at Chontas (0.92), intermediate for the regional network and Higuerón (both 0.75), and lowest at Numparket (0.65). Comparisons should consider that the regional network aggregates interactions across sites, which can shift metric values relative to any single local network.

Navigate back to Table 3.

## Table 4 Long description

The table reports pairwise dissimilarity between interaction networks at three site pairs, separating overall dissimilarity (βWN) into rewiring among shared species (βOS) and species turnover (βST), with βST further split into fly-only (βST.f), bat-only (βST.b), and joint turnover (βST.fb). Rewiring is identical and low for all comparisons (βOS = 0.04). Overall dissimilarity (βWN) ranges from 0.57 to 0.70 and is driven mainly by turnover (βST = 0.54–0.67). Chontas–Numparket has the greatest βWN (0.70) and βST (0.67), with turnover components 0.22 (fly), 0.07 (bat), and 0.37 (joint). Higueron–Numparket has the lowest βWN (0.57) and βST (0.54), with bat-only turnover at 0 and joint turnover at 0.32. Higueron–Chontas is intermediate (βWN 0.60; βST 0.56), with joint turnover (0.28) larger than fly-only (0.16) or bat-only (0.12). Values are unitless indices; differences indicate relative dissimilarity but do not convey statistical uncertainty.

Navigate back to Table 4.
