# Supplementary material for: Specialization of bat-fly interactions at different elevations in a montane forest of northern Peru
Source: Parasitology. 2025 Dec 26;153(3):352–65. doi: 10.1017/S0031182025101479 (PMC13215737; doi:10.1017/S0031182025101479)
Supplement: Minaya et al. supplementary material [file S0031182025101479sup001.docx]

Supplementary table S1. Values of d' specialization for all bat and fly species.

| **Organism** | **Species** | **Regional network** | **Numparket**  **(1800 m)** | **Chontas**  **(1560 m)** | **Higueron**  **(1480 m)** |
| --- | --- | --- | --- | --- | --- |
| Fly | *Exastion deceptivum* | 0.83 | 0.77 | NA | NA |
| Fly | *Exastion oculatum* | 0.94 | 0.92 | NA | NA |
| Fly | *Neotrichobius bisetosus* | 0.89 | NA | 1 | 0.84 |
| Fly | *Aspidoptera phyllostomatis* | 0.94 | NA | NA | 0.91 |
| Fly | *Metelasmus pseudopterus* | 0.56 | NA | NA | 0.4 |
| Fly | *Paraeuctenodes similis* | 0.39 | 0.33 | 0.57 | 0.35 |
| Fly | *Strebla guajiro* | 0.05 | 0 | NA | 0 |
| Fly | *Trichobius joblingi* | 0.38 | 0.23 | 0.65 | 0.41 |
| Fly | *Anastrebla caudiferae* | 0.24 | 0.21 | NA | NA |
| Fly | *Speiseria ambigua* | 0.24 | NA | 0.39 | 0.27 |
| Fly | *Anastrebla* sp. | 1 | 1 | NA | NA |
| Fly | *Anatrichobius scorzai* | 0.67 | NA | 0.61 | 0.65 |
| Fly | *Basilia anceps* | 0.66 | NA | 0.69 | NA |
| Fly | *Basilia* sp. | 0.57 | NA | NA | 0.78 |
| Fly | *Joblingia schmidti* | 0.37 | 1 | NA | NA |
| Fly | *Paratrichobius longicrus* | 1 | 1 | NA | NA |
| Fly | *Megistopoda proxima* | 0.93 | 1 | 0.85 | NA |
| Fly | *Aspidoptera delatorrei* | 0.87 | NA | 0.82 | NA |
| Fly | *Trichobius petersoni* | 0.82 | 1 | NA | 0.63 |
| Bat | *Anoura aequatoris* | 0.89 | 0.86 | NA | NA |
| Bat | *Anoura peruana* | 0.94 | 0.91 | NA | NA |
| Bat | *Artibeus glaucus* | 0.82 | NA | 1 | NA |
| Bat | *Artibeus planirostris* | 1 | NA | NA | 1 |
| Bat | *Carollia brevicauda* | 0.54 | 0.36 | 0.8 | 0.56 |
| Bat | *Carollia perspicillata* | 0.36 | 0.35 | 0.62 | 0.28 |
| Bat | *Choeroniscus minor* | 1 | 1 | NA | NA |
| Bat | *Myotis nigricans* | 0.73 | NA | 0.68 | NA |
| Bat | *Myotis riparius* | 0.79 | 1 | 0.6 | 1 |
| Bat | *Platyrrhinus fusciventris* | 1 | 1 | NA | NA |
| Bat | *Sturnira bidens* | 0.32 | 0.47 | NA | NA |
| Bat | *Sturnira oporaphilum* | 0.89 | 0.9 | 0.86 | NA |
| Bat | *Sturnira tildae* | 0.78 | NA | 0.78 | NA |
| Bat | *Vampyrodes caraccioli* | 0.89 | 1 | NA | 1 |

Supplementary table S2. Values of species strength for all bats and fly species.

| **Organism** | **Species** | **Regional network** | **Numparket**  **(1800 m)** | **Chontas**  **(1560 m)** | **Higueron**  **(1480 m)** |
| --- | --- | --- | --- | --- | --- |
| Fly | *Exastion deceptivum* | 1.17 | 1.17 | NA | NA |
| Fly | *Exastion oculatum* | 0.83 | 0.83 | NA | NA |
| Fly | *Neotrichobius bisetosus* | 1.5 | NA | 1 | 0.67 |
| Fly | *Aspidoptera phyllostomatis* | 0.83 | NA | NA | 0.83 |
| Fly | *Metelasmus pseudopterus* | 0.17 | NA | NA | 0.17 |
| Fly | *Paraeuctenodes similis* | 0.91 | 1.07 | 1.25 | 0.56 |
| Fly | *Strebla guajiro* | 0.07 | 0.05 | NA | 0.16 |
| Fly | *Trichobius joblingi* | 0.91 | 0.79 | 0.58 | 1.17 |
| Fly | *Anastrebla caudiferae* | 0.06 | 0.1 | NA | NA |
| Fly | *Speiseria ambigua* | 0.06 | NA | 0.17 | 0.11 |
| Fly | *Anastrebla* sp. | 1 | 1 | NA | NA |
| Fly | *Anatrichobius scorzai* | 0.86 | NA | 0.84 | 0.4 |
| Fly | *Basilia anceps* | 0.83 | NA | 1.16 | NA |
| Fly | *Basilia* sp. | 0.23 | NA | NA | 0.6 |
| Fly | *Joblingia schmidti* | 0.08 | 1 | NA | NA |
| Fly | *Paratrichobius longicrus* | 1 | 1 | NA | NA |
| Fly | *Megistopoda proxima* | 2.33 | 2 | 1.33 | NA |
| Fly | *Aspidoptera delatorrei* | 0.67 | NA | 0.67 | NA |
| Fly | *Trichobius petersoni* | 0.5 | 1 | NA | 0.33 |
| Bat | *Anoura aequatoris* | 0.67 | 0.67 | NA | NA |
| Bat | *Anoura peruana* | 1.33 | 1.33 | NA | NA |
| Bat | *Artibeus glaucus* | 0.5 | NA | 1 | NA |
| Bat | *Artibeus planirostris* | 2 | NA | NA | 2 |
| Bat | *Carollia brevicauda* | 1.64 | 0.99 | 1.62 | 2.03 |
| Bat | *Carollia perspicillata* | 3.36 | 3.01 | 1.38 | 1.98 |
| Bat | *Choeroniscus minor* | 1 | 1 | NA | NA |
| Bat | *Myotis nigricans* | 1 | NA | 1.16 | NA |
| Bat | *Myotis riparius* | 3 | 1 | 0.84 | 2 |
| Bat | *Platyrrhinus fusciventris* | 1 | 1 | NA | NA |
| Bat | *Sturnira bidens* | 0.08 | 0.2 | NA | NA |
| Bat | *Sturnira oporaphilum* | 0.77 | 0.8 | 0.75 | NA |
| Bat | *Sturnira tildae* | 1.15 | NA | 1.25 | NA |
| Bat | *Vampyrodes caraccioli* | 1.5 | 1 | NA | 2 |
